# First episode depression during the perinatal period is associated with atopic diseases and persistently increased eosinophil and basophil levels

**DOI:** 10.1007/s00737-024-01522-5

**Published:** 2024-10-16

**Authors:** En-Young N. Wagner, Eva Maria Pichler, Mario Müller, Andrea Eisenhut, Ana Buadze, Yanhua Xu, Erich Seifritz, Marie-Pierre F. Strippoli, Enrique Castelao, Setareh Ranjbar, Jennifer Glaus, Caroline Vandeleur, Martin Preisig, Roland von Känel, Vladeta Ajdacic-Gross

**Affiliations:** 1 Department of Consultation-Liaison Psychiatry and Psychosomatic Medicine, University Hospital Zurich, University of Zurich, Haldenbachstrasse 16/18, CH-8091 Zurich, Switzerland; 2 Department of Psychiatry and Psychotherapy, Psychiatric Services Aargau, Windisch, Switzerland; 3 Department of Adult Psychiatry and Psychotherapy, Psychiatric University Clinic Zurich, University of Zurich, Zurich, Switzerland; 4 Department of Psychiatry, Psychiatric Epidemiology and Psychopathology Research Center, Lausanne University Hospital, University of Lausanne, Prilly, Switzerland; 5 Department of Psychiatry, Service of Child and Adolescent Psychiatry, Lausanne University Hospital, University of Lausanne, Lausanne, Switzerland

**Keywords:** Perinatal, Depression, Atopy, Eosinophils, Basophils

## Abstract

**Purpose:**

A previous diagnosis of depression is a strong predictor for perinatal depression, apart from other mental disorders, stress, and atopies. It is less clear which factors interfere if perinatal depression occurs as a first depression episode (fePND).

**Methods:**

We examined the associations with atopies and related blood parameters using data of CoLaus|PsyCoLaus.

**Results:**

Newly occurring depression during the perinatal period but not recurrent depression was associated with a lifetime diagnosis of allergies and asthma together with persistently increased levels of basophils and eosinophils.

**Conclusion:**

The results imply that immune function may play a relevant role in the risk of a fePND. If confirmed and detailed, these findings could serve as the basis for designing preliminary prevention strategies by observing eosinophil and basophil levels as well as symptoms of atopic diseases before/during pregnancy.

**Supplementary Information:**

The online version contains supplementary material available at 10.1007/s00737-024-01522-5.

## Introduction

Perinatal depression (PND) occurs in up to 15% of all parturients, and the risk of recurrence is high, with a 15–21% prevalence in women without prior psychiatric disorder (Rasmussen et al. 2017). Nevertheless, there is growing evidence that PND should be distinguished from other depressive disorders (Sherer et al. 2018). While the main predictor for PND is a depressive episode in the history (Gastaldon et al. 2022), little is known about the factors associated with a first episode of depression during the perinatal period. If PND were actually different from other subtypes of depression, then first episode PND (fePND) – rather than subsequent episode PND (sePND) – would deserve renewed attention.

Pregnancy brings with it fundamental bodily changes comprising altered hormonal and immune processes, but also including an increased plasticity and restructuring of the brain (Sherer et al. 2018). Periods of increased plasticity are at the same time periods of increased brain vulnerability (Tesic et al. 2019), not least induced by diverse pro-inflammatory processes in the periphery. Atopic diseases and related immune processes would seem to play a prominent role in this connection. Recent findings have indicated that a history of allergic asthma, rhinitis and/or dermatitis in pregnant women is associated with a higher risk of developing PND (Blais et al. 2019; Aker et al. 2022). In this study, we aimed to extend current knowledge about the associations between the atopies and PND in two directions. The first direction was to look for differences in associations between sePND and fePND. The second was to examine eosinophil and basophil levels, given that higher levels are typically associated with atopies, remaining persistently elevated therafter (Ajdacic-Gross et al. 2019). Thus, we hypothesized that if PND were associated with atopies, this would be primarily fePND and would accompany persistently increased eosinophil and basophil levels. The analysis was based on data from the CoLaus|PsyCoLaus study.

## Methods

CoLaus|PsyCoLaus is a large interdisciplinary population-based epidemiological study conducted in Lausanne, Switzerland. It was designed to investigate cardiovascular risk factors and mental disorders in the community and to determine their associations. Its reach goes far beyond the initial focus, however. Due to the wealth of information and range of physiological markers, it has been particularly prolific for joint analyses of somatic and mental conditions.

CoLaus|PsyCoLaus (Firmann et al. 2008; Preisig et al. 2009) was designed as a prospective cohort study. The initial sample (*n* = 6,734) was randomly selected among the 35- to 75-year-old residents of the city of Lausanne (Switzerland) according to the civil register. After a first physical assessment, which took place between 2003 and 2008, the cohort was followed-up approximately 5 years (Follow-up 1, FU1) and 10 years (Follow-up 2, FU2) later. For most of the participants, the psychiatric assessment started at baseline (*N* = 3,719); at FU1, *N* = 1,155 new assessments were contributed and at FU2 *N* = 237 (see more detailed description in Supplementary material). While the somatic part of the study (CoLaus) favors longitudinal analyses, the psychiatric part (PsyCoLaus) mostly relies on cross-sectional analyses of self-reported retrospective information from baseline assessments. This difference is understandable in view of the initial age range, and also applies to the current analysis. Among the 5,111 participants with a psychiatric evaluation, 2,741 were women, and 2,074 of them mothers who reported giving birth to at least one child.

The French version (Preisig et al. 1999) of the semi-structured Diagnostic Interview for Genetic Studies (DIGS) (Nurnberger et al. 1994) was used to assess diagnostic information on mental disorders, including major depressive disorder (MDD) according to the Diagnostic and Statistical Manual of Mental Disorders Fourth edition (DSM-IV-TR) (American Psychiatric Association 2013). The diagnostic information is retrospective, referring to a whole lifetime. Interviews were conducted by trained psychologists.

PND was determined in the depression section of the DIGS. In addition to MDD criteria, the following question was asked: “Did this episode occur at the time of giving birth to a child?“. Moreover, all relevant comment variables from the depression section were checked for additional information. Exclusion criteria were potentially traumatic experiences due to miscarriage, death of the newborn or serious disease/ handicap of the newborn. Furthermore, PND was differentiated into two categories, depending on whether it was the first major depressive episode (fePND) or a subsequent episode (sePND). Technically, fePND was restricted to the same year as the onset of MDD.

Information on socio-demographic characteristics comprised the number of deliveries, which was grouped into 3 categories: 1, 2, 3 or more births. Age at delivery was assessed in connection with PND and compared between fePND and sePND respondents. Socioeconomic status was assessed with the Hollingshead index (Hollingshead 1975) based on marital status, educational achievement and employment status.

The information on atopic diseases was collected using an extended version of the medical history parts of the DIGS used in the psychiatric assessment, and was based on self-reporting. The questions on the medical history covered life-time prevalence and were followed-up by information on age of onset of each disease answered in the affirmative.

Blood samples were taken at each somatic assessment (baseline, FU1, FU2). Morning venous blood samples served to assess eosinophil and basophil counts, white blood cell (WBC) counts and further markers in CoLaus|PsyCoLaus. The assessment of these markers is described in detail elsewhere (Firmann et al. 2008; Ajdacic-Gross et al. 2021). The eosinophil and basophil counts were determined at FU1 and FU2. Given the age range of the cohort, this mostly occurred years after giving birth to a child, which implies that the focus was on continuingly shifted eosinophil and basophil levels. The availability of double (or more generally: multiple) measurements is a great advantage in view of this focus.

For a detailed description of the data preprocessing steps see (Ajdacic-Gross et al. 2021). Briefly, after checking normal distribution (Kolmogorov-Smirnov and Shapiro-Wilk test) the eosinophil and basophil values were transformed by square root. Outlier values (< / > 3 standard deviations) were set to missing. To use averaged values from data from two measurements – if available – the variables were submitted to z-transformation and were age-adjusted using linear regression.

The data were preprocessed with SPSS version 28 and analyzed with basic statistical procedures. Descriptive statistics were provided in terms of frequencies and means with standard errors for overall PND, sePND and fePND. In analyses with categorical variables (asthma, hay fever), odds ratios of PND, sePND and fePND were derived from contingency tables. In analyses with metric variables (basophils, eosinophils) we used the t-test.

## Results

Overall, 60 out of 2074 mothers in the CoLaus|PsyCoLaus study had had a major depressive disorder occurring around the birth of a child. Of these, 34 were classified as fePND and 26 as sePND.

The socio-economic status as measured by the Hollingshead index with five categories did not significantly differ between these two groups (chi2 = 6.09, d.f. 4, *p* = .19), nor did it differ between mothers with and without PND (chi2 = 4.67, d.f. 4, *p* = .32). The same applies for the number of deliveries (3 categories: 1, 2, 3+) (PND vs. other: chi2 = 0.68, d.f. 2, *p* = .79; fePND vs. sePND: chi2 = 4.19, d.f. 2, *p* = .12). The age at delivery of women with fePND was significantly lower than in women with sePND: 28.2y (SE 0.8) vs. 33.2y (SE 1.3, *p* = .001).

Based on contingency tables, women with lifetime asthma and/or hay fever had > 2-fold higher odds of reporting fePND (Table 1, ORs). No associations were found between these variables in terms of sePND.

**Table 1:** Associations between perinatal depression and atopic diseases; frequencies (with proportions) and odds ratios derived from contingency tables

	no PND	PND	OR (CI)	sePND	OR (CI)	fePND	OR(CI)
	(*N *= 2014)	(*N *= 60)		(*N *= 26)		(*N *= 34)	
asthma	263 (13.1%)	13 (21.7%)	1.84 (0.98–3.45)	4 (15.4%)	1.19 (0.41-3.47)	9 (26.5%)	2.39 (1.10–5.18)
hay fever	367 (18.2%)	15 (25.0%)	1.50 (0.83-2.71)	3 (11.5%)	0.57 (0.17-1.92)	12 (35.3%)	2.46 (1.21-5.02)

*PND *perinatal depression, *sePND *subsequent PND, *fePND *first episode PND, *OR * odds ratio, *CI *confidence interval

A similar pattern of associations emerged between PND and counts of basophils and eosinophils (Table 2.). Both types of white blood cell showed increased levels in respondents with PND. However, basophils had outstandingly high levels particularly in fePND, whereas eosinophils had similarly increased levels in both fePND and sePND respondents.

**Table 2.** Basophil and eosinophil levels in perinatal depression, overall and for subgroups; t-tests based on z-standardized and age-adjusted values

	*n*	all PND		sePND		fePND	
	PND / no PND	mean (SE)	*p*-value	mean (SE)	*p*-value	mean (SE)	*p*-value
basophils	55 / 1847	0.219 (0.121)	0.039	0.075 (0.216)	0.578	0.347 (0.125)	0.022
eosinophils	55 / 1836	0.252 (0.120)	0.027	0.238 (0.168)	0.155	0.264 (0.172)	0.096

*PND * perinatal depression, *sePND * subsequent PND, *fePND * first episode PND,* SE  *standard error

Notes: Basophils and eosinophils represented by z-standardized and age-adjusted values averaged from up to two measurements The mean-values represent deviations of these values from 0 Two-sided *p*-values derived from t-tests Differences compared to total n are due to missing values in blood samples The means of mothers without PND are ≅0 (due to z-standardization), SE≤0.018

## Discussion

This study examined the associations of PND with atopic diseases and related blood markers in a large Swiss community-based sample. The results marked a major difference between PND occurring as fePND and sePND. A lifetime history of atopic disease (asthma, hay fever) was associated exclusively with fePND. In contrast, no such associations could be shown for women with sePND. A similar configuration emerged with respect to basophil levels. fePND but not sePND was associated with continuingly increased levels of basophils, while both subgroups showed increased eosinophil levels.

Research on the association between PND and atopy is scarce. In a sample of 937,422 Danish women, those with allergic asthma/ rhinitis and/or atopic dermatitis before conception had a 37% increased overall risk of developing a mental disorder peripartum (Ren et al. 2021). In another study, women with asthma were more likely to develop PND as compared to women without asthma (6.1% versus 2.9%) (Blais et al. 2019). Furthermore, a higher grade of asthma exacerbation has been shown to increase the risk for PND in a cohort of 62,583 women (Aker et al. 2022).

While the associations between atopic diseases and mood disorders have been investigated for some time now (Goodwin et al. 2004), this study extends current knowledge to risk factors for the development of PND (Gastaldon et al. 2022) by showing that fePND but not sePND is associated with atopy and continuingly increased levels of both basophils and eosinophils. Increased levels of basophils, which are key defense cells involved in the development and progression of allergic inflammation (Siracusa et al. 2013), might deserve particular attention owing to the present results.

The theoretical framework of this study rests on two pillars. Atopic diseases come along with a disregulated activity of the immune system, reflected among other things by increased eosinophil and basophil levels, upregulated Th2 helper cells and related cytokine release (Folci et al. 2021). As with other peripheral inflammatory processes, they also impact the activity of microglia and other immunologically active brain cells (Chua et al. 2020; Tamayo et al. 2024), thus indirectly affecting brain functioning. This impact extends to brain development and restructuring, particularly during critical windows of development (e.g., first years of life, adolescence / young adulthood), thus increasing the vulnerability for neurodevelopmental disorders and mental disorders in an explicit age-specific manner (Tesic et al. 2019). Both pillars – a shift towards Th2-related immunity and increased brain plasticity during a critical time window – are also apparent in pregnancy (Sherer et al. 2018; Duarte-Guterman et al. 2019). Both are beneficial for the fetus and a successful pregnancy, but also bear specific risks as reflected by fePND.

The retrospective and exploratory design of the study provide limited information regarding the timing and the temporal interrelations between the variables of interest. It was not possible to determine the timing of the increase in eosinophil and basophil levels, which could have occurred before or in parallel to PND, in rare instances also after PND. From the perspective of basophils and eosinophils, two aspects are important: first, they are assumed to be permanently increased, so that the timing of the assessment is less relevant, and second, they are assumed to be related to a vulnerability for atopic diseases, i.e., initial immune processes, rather than to the manifest onset of the diseases. The manifest onset of hay fever or asthma is not unequivocally linked to the timing of emerging vulnerability in hay fever / asthma history and can occur after a long preclinical period. In sePND, increased eosinophil levels could represent PND, but they could also be associated with previous depression episodes. Different risk mechanisms for PND can be assumed to co-exist:

- Pre-existing atopy enhanced during pregnancy;
- Manifest atopic disease newly emerging during pregnancy;
- Vulnerability for atopy emerging during pregnancy and introducing a prodromal phase.

Any of these is hypothesized to imply consequences for imperfect brain restructuring during pregnancy. Large scale prospective studies, which could cover these uncertainties, are not feasible. Therefore, cross-sectional studies help to fill the gap despite their diverse limitations.

The common limitation of this study is its retrospective design with well-known biasing effects (recall bias, telescoping effect). At the same time, the retrospective design is the main strength, since it represents the only realistic way of bringing together the necessary information from all different age stages. Assessing PND via a major depressive episode yields distinctly lower figures than scales such as the Edinburgh Postnatal Depression Scale (Cox et al. 1987; Lyubenova et al. 2021). In addition, the frequencies were diminished by narrowing the time frame of the depressive episode to the same year as the delivery and by introducing exclusion criteria (miscarriage and other potentially traumatic experiences). Altogether this means that we see here only the tip of an iceberg and accordingly only associations related to the tip. More in-depth analyses were not recommendable, given the small sample of mothers with PND in this study.

To sum up, this study showed intriguing associations of fePND with atopies and persistently increased eosinophil / basophil levels in a community-based sample. FePND and sePND emerged as different depression subtypes. The results imply that immune function may play a relevant role in the risk of fePND, either preceding or reflecting the perinatal period. If confirmed and detailed, these findings could serve as the basis for designing preliminary prevention strategies by observing eosinophil and basophil levels as well as symptoms of atopic diseases before / during pregnancy. In any case, regular psychological assessments during pregnancy can facilitate early intervention, improving outcomes for mothers as well as newborns.

## Electronic supplementary material

Below is the link to the electronic supplementary material.

Supplementary Material 1 (DOCX 102 KB)
