# Supplementary material for: First episode depression during the perinatal period is associated with atopic diseases and persistently increased eosinophil and basophil levels
Source: Arch Womens Ment Health. 2024 Oct 16;28(3):639–44. doi: 10.1007/s00737-024-01522-5 (PMC12092543; doi:10.1007/s00737-024-01522-5)
Supplement: Supplementary file 1 — Supplementary Material 1 (DOCX 102 KB) [file 737_2024_1522_MOESM1_ESM.docx]

**Supplementary material 1:**

**The CoLaus|PsyCoLaus cohort**

Data used for the current study were derived from the population-based cohort of CoLaus|PsyCoLaus (Firmann, Mayor et al. 2008; Preisig, Waeber et al. 2009). The CoLaus|PsyCoLaus study was designed to explore associations between mental disorders and cardiovascular diseases. The cohort was randomly selected from the 35- to 75-year-old residents of the city of Lausanne (Switzerland) from 2003 to 2006 according to the civil register. Figure S1 displays the overall workflow of the sampling process. The initial cohort included 6,734 individuals. The first follow-up (FU1) was carried out from 2009-2013 and the second follow-up (FU2) was conducted from 2014-2018.

At baseline, the psychiatric evaluation, carried out by trained psychologists, was restricted to the 35- to 67-year-old participants in the physical exam, resulting in a 67% participation rate within this age range (N=3,719). From FU1 on, all individuals from the initial cohort were eligible for the psychiatric evaluation. The cohort used in the present paper is comprised of the first assessment of all 5,111 people (35 to 88 years old) who agreed to participate in at least one psychiatric evaluation (N=3,719 at baseline, N=1,155 at FU1, N=237 at FU2).

*Instrument in psychiatric assessment: DIGS*

The French version (Preisig, Fenton et al. 1999) of the semi-structured Diagnostic Interview for Genetic Studies (DIGS) (Nurnberger, Blehar et al. 1994), was used to collect diagnostic information on mental disorders. The assessments covered a broad spectrum of the DSM-IV Axis I criteria, as well as the course and chronology of comorbid features. The French version of the DIGS revealed excellent inter-rater reliability in terms of kappa and Yule's Y coefficients for major mood and psychotic disorders (Preisig, Fenton et al. 1999)as well as for substance use and antisocial personality disorder (Berney, Preisig et al. 2002), whereas the 6-week test-retest reliability was slightly lower (Berney, Preisig et al. 2002).

Figure S1: The overall workflow of the CoLaus|PsyCoLaus sampling.

**Second survey
2009-2013**

**Third survey
2014-2018**

**First survey
2003-2007**

First physical exam

age 35-75 years

n = 6733

CoLaus¦PsyCoLaus

age 35-75 years

n = 6734

No participation in psychiatric exam

No participation in psychiatric exam

First psychiatric exam

age 35-66 years

n = 3719

First psychiatric exam

age 41-81 years

n = 1155

First psychiatric exam

age 36-86 years

n = 237

First psychiatric exam of participants from first, second or third survey

n = 5111

**Supplementary Material 2:**

**Somatic assessments**

Data on somatic conditions and physiological markers were taken either from the medical section of the psychiatric interview or from the physical (CoLaus) assessments that had taken place prior to the psychiatric assessments. Morning venous blood samples served to assess WBC counts and other physiological markers. WBC counts of neutrophils, lymphocytes, monocytes, eosinophils and basophils were computed both as absolute values and as proportions of the total WBC count. The assessment of physiological markers is described in detail elsewhere (Firmann, Mayor et al. 2008; Marques-Vidal, Bochud et al. 2011; Ajdacic-Gross, Ajdacic et al. 2021). The markers were assessed up to three times, i.e., at the baseline examination and at the follow-up 1 and 2 evaluations. The WBC counts were determined only at the follow-ups.

In pre-processing the physiological marker data, the Kolmogorov-Smirnov and the Shapiro-Wilk test served to check for the normal distribution of the marker variables. If appropriate, the variables were transformed by log or square root. Values were considered as outliers if above / below 3 standard deviations (SD) and were set to missing. In the next step, the variables were z-transformed. This enabled the averaging of the variables if data from two or all three measurements were available. Finally, the variables were age-standardized using a linear or quadratic regression, if appropriate, to exclude bias related to different age patterns of marker variables (referring to the age at measurement) (Ajdacic-Gross, Ajdacic et al. 2021).

**Supplementary material 3:**

**Raw values of basophils / eosinophils by measurement, overall sample, women**

baseline measurement 1st follow-up measurement 2nd follow-up measurement units data transformation ^1^

N mean SE N mean SE N mean SE

basophils - - - 1667 0.036 0.0005 2179 0.047 0.0007 G/l sq

eosinophils - - - 1667 0.166 0.0027 2178 0.164 0.0026 G/l sq

**References**

Ajdacic-Gross, V, Ajdacic, L, Xu, Y, Müller, M, Rodgers, S, Wyss, C, Olbrich, S, Buadze, A, Seifritz, E, Wagner, EN, Radovanovic, D, von Wyl, V, Steinemann, N, Landolt, MA, Castelao, E, Strippoli, MF, Gholamrezaee, MM, Glaus, J, Vandeleur, C, Preisig, M, von Känel, R (2021) Backtracing persistent biomarker shifts to the age of onset: A novel procedure applied to men’s and women’s white blood cell counts in post-traumatic stress disorder. Biomarkers in Neuropsychiatry 4: 1-10. <https://doi.org/10.1016/j.bionps.2021.100030>

Berney, A, Preisig, M, Matthey, ML, Ferrero, F, Fenton, BT (2002) Diagnostic interview for genetic studies (DIGS): inter-rater and test-retest reliability of alcohol and drug diagnoses. Drug Alcohol Depend 65: 149-158. <https://doi.org/>

Firmann, M, Mayor, V, Vidal, PM, Bochud, M, Pecoud, A, Hayoz, D, Paccaud, F, Preisig, M, Song, KS, Yuan, X, Danoff, TM, Stirnadel, HA, Waterworth, D, Mooser, V, Waeber, G, Vollenweider, P (2008) The CoLaus study: a population-based study to investigate the epidemiology and genetic determinants of cardiovascular risk factors and metabolic syndrome. BMC Cardiovasc Disord 8: 6. <https://doi.org/10.1186/1471-2261-8-6>

Marques-Vidal, P, Bochud, M, Bastardot, F, Luscher, T, Ferrero, F, Gaspoz, JM, Paccaud, F, Urwyler, A, von Kanel, R, Hock, C, Waeber, G, Preisig, M, Vollenweider, P (2011) Levels and determinants of inflammatory biomarkers in a Swiss population-based sample (CoLaus study). PLoS One 6: e21002. <https://doi.org/10.1371/journal.pone.0021002>

Nurnberger, JI, Jr., Blehar, MC, Kaufmann, CA, York-Cooler, C, Simpson, SG, Harkavy-Friedman, J, Severe, JB, Malaspina, D, Reich, T (1994) Diagnostic interview for genetic studies. Rationale, unique features, and training. NIMH Genetics Initiative. Arch Gen Psychiatry 51: 849-859. <https://doi.org/>

Preisig, M, Fenton, BT, Matthey, ML, Berney, A, Ferrero, F (1999) Diagnostic interview for genetic studies (DIGS): inter-rater and test-retest reliability of the French version. Eur Arch Psychiatry Clin Neurosci 249: 174-179. <https://doi.org/>

Preisig, M, Waeber, G, Vollenweider, P, Bovet, P, Rothen, S, Vandeleur, C, Guex, P, Middleton, L, Waterworth, D, Mooser, V, Tozzi, F, Muglia, P (2009) The PsyCoLaus study: methodology and characteristics of the sample of a population-based survey on psychiatric disorders and their association with genetic and cardiovascular risk factors. BMC Psychiatry 9: 9. <https://doi.org/10.1186/1471-244X-9-9>
